# Global autism prevalence, and exploring Montessori as a practical educational solution: a systematic review

**DOI:** 10.3389/fpsyt.2025.1604937

**Published:** 2025-06-16

**Authors:** Ying Li

**Affiliations:** Faculty of Human Development, University Pendidikan Sultan Idris, Perak, Malaysia

**Keywords:** autism spectrum disorder, ASD prevalence, ASD early intervention, ASD educational solutions, Montessori, ASD behavioral therapy, ASD pharmacological treatment, artificial intelligence in ASD

## Abstract

**Introduction:**

Autism spectrum disorder (ASD) is a neurodevelopmental condition that impairs social interaction, communication, and appropriate behavior.

**Methods:**

Out of 1,740 articles initially identified through electronic databases using PRISMA guidelines and the PICOS framework, only 15 met the inclusion criteria for this review.

**Results:**

Although the precise etiology of autism remains unclear in most cases, from cohort studies, the heritability as a potential cause is estimated to range between 50% and 80%, taking into account consistent environmental risk factors such as parental age, pollution, and maternal infections during pregnancy. Several autism-related gene mutations have also been identified. Recommended interventions include applied behavior analysis, speech therapy, pharmacological treatment, and emerging techniques such as artificial intelligence, virtual reality, and microbiome-based approaches. Pharmacological agents like Risperidone and Aripiprazole can alleviate specific symptoms but do not target the core features of ASD. Additional evidence-based therapies, including occupational therapy and sensory integration, have demonstrated benefits in enhancing functional outcomes.

**Discussion:**

Montessori education, a sensory-focused, individualized, and play-based learning approach, aligns well with the individual learning needs of ASD and shows considerable potential in improving communication and social skills in children with ASD. This systematic review underscores geographic disparities and risk factors associated with autism while uniquely assessing Montessori education as a promising intervention, paving the way for further research in low-resource settings.

## 1 Introduction

Autism Spectrum Disorder (ASD) is a developmental disorder impacting social interaction, communication, and the presentation of repetitive behaviors or intensely fixated interests. It is not evenly spread between males and females and tends to manifest in very early years. Although the manifestation and type can be different, ASD is, at its core, a condition that impairs a person’s way of interacting with others and their inability to cope with the environment (1). In the last 20 years, more people, both domestically and internationally, have been diagnosed with ASD, and the epidemiology, etiology, and treatment of ASD have become emerging research focuses.

### 1.1 ASD global overview

The WHO estimates that 1 in every 160 children in the world have ASD (2), whereas, according to CDC, about 1 in every 36 children who are 8 years old in the United States were diagnosed with ASD in 2020, a prevalence that was as 1 in 150 in 2000 (3). These increases are attributed to advancements in diagnostic criteria, increased awareness, and enhanced screening/surveillance systems (4, 5). The 2013 shift from DSM-IV to DSM-5 revised diagnostic thresholds by grouping disorders like Asperger’s syndrome and PDD-NOS into ASD, potentially contributing to increased prevalence (6, 7). However, these changes also posed diagnostic difficulties because females and adults are often paucisymptomatic and are underdiagnosed (8, 9). Past criteria have proved to be wanting when identifying high-functioning persons and females, as they are likely to feign or exhibit symptoms dissimilar to those of male patients (10).

### 1.2 Genetic and environmental factors

Scientific information has strengthened the evidence for the role of genetic factors in the development of ASD, with twin studies suggesting a heritability index of between 50%-80% (11). Studies have pointed out that ASD has connections with several gene mutations and copy number variations like the SHANK3, CHD8, NRXN1, and FMR1 (12, 13). These mutations impact synaptic transmission, neuronal growth, and structural plasticity, essential for cognitive and behavioral control. DNA methylation and histone modification facilitate gene-environment interactions (epigenetic mechanisms), mediating the impact of environmental factors on genetic predispositions during neural development, potentially contributing to ASD’s connectivity and learning deficits (14). Other research indicates that epigenetic mechanisms may play a role in the disruption of connectivity and learning deficits in children with ASD (15).

Environmental risks are also very influential in the development of this ASD. Previous prenatal factors, including greater maternal age, paternal age, Gestational Diabetes Mellitus (GDM), preterm birth, cesarean section, and low birth weight, have been categorized as prenatal and perinatal risk factors for ASD (16–18). Airborne pollutants such as nitrogen dioxide and particulate matter, metals including mercury and lead, pesticides, and endocrine-disrupting chemicals are other environmental factors that have been shown to adversely affect neurodevelopment and influence the likelihood of ASD development (19–21). One notable factor is Maternal Immune Activation (MIA), which occurs when the mother’s immune system is triggered by increasing factors such as infection, autoimmune diseases, or inflammation during pregnancy. MIA has been demonstrated in animal and human studies to disrupt fetal brain development (21, 22). Children with ASD may have low vitamin D, B6, B12, folates, zinc, magnesium, and omega-3 fatty acids, which are crucial for neurotransmitter synthesis and immune function (23).

Emerging trends indicate that Maternal Metabolic Outcomes (MMO) such as GDM, obesity, and preeclampsia augment the odds of ASD in offspring (24). These disruptions are believed to trigger inflammatory responses, oxidative stress, and hormonal imbalances, which can negatively impact fetal brain development (25). For example, maternal diabetes, especially during the third trimester, may affect brain plasticity and synaptogenesis due to hyperglycemia and insulin resistance (2).

### 1.3 ASD diagnostic challenges

There are striking differences in ASD diagnosis and further management of the disorder regarding the region and socioeconomic status (SES). ASD is often undiagnosed in low- and middle-income countries (LMICs) because the healthcare systems are scanty; there is a dearth of specialists to attend to these patients. Society has a nondescriptive attitude towards mental health disorders (26). For instance, research on sub-Saharan African and South-Asian countries makes lower prevalence rate estimates, which might have been influenced by under-diagnosis instead of under-incidence (27, 28). In many such regions, children can end up being diagnosed with intellectual disability or psychiatric disorders when, in truth, they have ASD because of inadequate culturally sensitive screening tools (29). Racial and ethnic minorities, in particular, are documented to receive a diagnosis and are provided early intervention services later than others in high-income countries (30, 31). These disparities are attributed to language solutions that act as barriers, disparate healthcare access, and institutional racism that hinder good diagnosing and early intervention.

New systems of picturing autism have proposed concepts such as the attribute LAZY that encompasses Late-diagnosed, Atypical, Zero-yield screen patients with ASD forms that are less likely to be diagnosed at an early age or are high-functioning or female (25). These may not present classical diagnostic signs during initial assessments performed during childhood, especially when the diagnostic instruments are not sensitive to gender or cultural differences. Reluctance to acknowledge “My child is Autistic,” especially in Arab countries, widens this gap. Recent studies indicate Qatar has a high ASD prevalence, with 2019 research estimating 1.4% among school-aged children (32) and another reporting 1.14% among children aged 6 to 11 years (33). Some communities attribute ASD behaviors to possession by evil spirits or witchcraft, delaying diagnosis and treatment.

M-CHAT and ADOS-2: Current large-scale screening methods used globally to identify the early signs of ASD include the Modified Checklist for Autism in Toddlers (M-CHAT) and the Autism Diagnostic Observation Schedule (ADOS-2) (34).

### 1.4 ASD- interventions

#### 1.4.1 Dietary interventions

Various dietary therapies like gluten-free casein-free (GFCF) diets have been tried with learners with AD/HD. Without a clear overall impact of elimination diets, some meta-analyses indicate positive results in social behavior, hyperactivity, and irritability when dietary changes are sustained and personalized (35). Randomized pellets have also shown minor language and social competency improvement through supplementation with Omega-3 fatty acids (36). These results have implications for nutritional screening and individualized supplementation as part of the comprehensive therapy of ASD. This is more so in LMICs, where nutrient deficiencies, together with systemic health and dietary accessibility challenges, already amplify symptoms of ASD (37, 38). Research shows that approximately 70% of children with ASD show gastrointestinal symptoms with irregular gut microbiota, known as the gut-brain axis, prompting speculation that microbes within the gut may play some role regarding behaviors (39, 40).

#### 1.4.2 Educational solutions

Children with autism can benefit from Montessori-based education because it provides structure Lillard. Montessori education is gradually gaining a reputation as an effective model for children with ASD because of its nature-based approach, use of sensory explorations, and promotion of practical life skills. The teachings inherent in the Montessori approach include freedom of choice and learning at one’s own pace, which is very valuable for autistic children because of their learning style preferences. The School for Asperger has been designed as a structured but very loose program in which children can interact through play with objects and people with different stimuli for their senses. Furthermore, focusing on fine motor skills and coordination increases the child’s independence, which is generally a concern for children with autism. In addition, it has been suggested that the Montessori classroom environment is highly social and allows for social and communication skills development during peer interactions. The fundamental characteristics of Montessori education, including the individualized approach, sensorial activities, and the focus on practical life, contribute to the effectiveness of the Montessori approach for children with ASD in the academic, social, and developmental domains (38).

#### 1.4.3 Pharmaceutical interventions

No drug is known to treat ASD, but the side-by-side use of medications like Risperidone and Aripiprazole may help control some symptoms such as anxiety, aggression, and repetitive actions (27).

#### 1.4.4 Behavioral and communication intervention

Other forms of intervention include ABA, speech therapy, and occupational therapy, which enhance communication and social interaction among ASD individuals (41).

#### 1.4.5 Other interventions solutions

Artificial Intelligence (AI) technologies such as diagnostic tools, Augmentative and Alternative Communication (AAC) devices, and Virtual Reality (VR) therapy can be regarded as innovations proposed for the treatment of autism (28).

### 1.5 Summary

However, the source of ASD is still not well understood due to its multi-faceted nature and the significant impact of the disorder on children all over the world; therefore, there is a need to compile knowledge about different patterns of prevalence and factors related to the occurrence of ASD as well as interventions for the disorder. There is growing interest in the use of educational approaches in the management of ASD, especially those that are individualized. One such is Montessori training, which is peaceful, systematic, touch-tangible, and student-centered. A couple of former studies highlight that Montessori approaches, such as autonomous discovery, handling things, and group work for a group, are consistent with learning styles preferred by children with ASD, especially regarding communication and behavior improvements (38). However, this promising approach has been used in systematic reviews but not explored in detail.

Hence, this review will compile a comprehensive synthesis of the global prevalence rate of ASD and explore genetic, epigenetic, and environmental factors that underlie the condition and existing intervention approaches with a special emphasis on the Montessori approach. This synthesis is drawn to help clinicians, educators, and policymakers make sense of the current state of ASD research and to identify future directions of care and research.

## 2 Materials and methods

### 2.1 Study design

This systematic review follows the Preferred Reporting Items for Systematic Reviews and Meta-Analyses (PRISMA) criteria to guide their conduct to be clear and precise. This review aimed to review literature documenting the prevalence of ASD worldwide, the associated genetic and environmental causes, and the effectiveness of the existing intervention methods with specific reference to Montessori education. No quantitative meta-synthesis was performed due to the imbalance and heterogeneity in some variables employed in the discerned studies that ranged in match utilities, interventions and outcomes measures, and region environment.

### 2.2 Selection criteria

The study selection process adhered to the PRISMA framework, involving four stages: identification, screening, eligibility, and inclusion. A total of 1,740 records were identified through 5 significant databases. The following selection criteria were applied: 1) answered the study question, 2) written in English, were published in peer-reviewed journals, 3) used rigorous methodology, 4) research indicating people with ASD of different age groups and genders from different geographic and socio-economic backgrounds, 5) Previous research on screening approaches to MMO in children and adults categorized the assessments into behavioral and pharmacological treatment perquisites, and/or policy-based and educational solutions were included, 6) researches that compare checklists, including the Autism Diagnostic Observation Schedule (ADOS-2) and Modified Checklist for Autism in Toddlers (M-CHAT), 7) targeted population was clearly defined with sample size greater than 50 cases and controls to give enough power to the study, and 8) the review only examined the articles published from the year 2000 up to 2024 to enhance the chances of covering the eventuality of capturing the recent knowledge in autism. Five analytical and epidemiological study types were considered for inclusion: cohort, cross-sectional, case-control, RCTs, and systematic reviews. The review also focused on those works reporting attempts to apply new or unique methods or adapt them to the regional particulars for those with autism, e.g., Montessori education. After applying the selection criteria, 480 were found to be duplicates, and 1,260 records were screened by title and abstract. Of these, 980 were excluded for irrelevance, leaving 280 full-text articles for eligibility assessment. Following the application of PICOS criteria, 265 articles were excluded due to lack of primary data, non-peer-reviewed status, small sample sizes (<50), or methodological limitations. Ultimately, 15 studies were included for qualitative synthesis and risk of bias assessment. The PRISMA flow chart, presented in the Results section, visually summarizes this process. The title and abstract screening steps were performed independently and blinded by two investigators to minimize bias. Only the full-text articles that met the initial criteria were evaluated in the second step.

### 2.3 Exclusion criteria

The excluded studies were: 1) non-peer-reviewed conferences, editorials, or opinion pieces and did not contain research data; 2) studies that had articles that were specific to comorbidity and did not research AUTISM or had it as one of the co-primary diagnoses were excluded, 3) Any study conducted before the year 2000 was excluded unless it based research on autism in any way, 4) Studies with a sample size (<50) participants were also not considered due to low statistical power, 5) The studies conducted in animals without any direct association with clinical diagnosis and management of ASD, and 6) studies that appeared in journals, books, or were otherwise published in a language other than English were excluded if no official translation was available.

## 3 Methodology

### 3.1 Search strategy

A comprehensive and systematic literature search was conducted across five significant databases: PubMed, Scopus, Web of Science, Cochrane Library, and Google Scholar. The search spanned publications from January 2000 to March 30, 2024. Boolean operators (AND, OR) and Medical Subject Headings (MeSH) were used to construct the search queries. Key terms included: “Autism Spectrum Disorder,” “ASD prevalence,” “autism diagnosis,” “genetic factors in ASD,” “environmental risk factors for autism,” “early intervention for ASD,” “Montessori education and autism,” and “behavioral therapy for autism.” The search strategy was refined repeatedly to maximize sensitivity and specificity. Grey literature and preprints were not included due to the scope of the review. The study was not preregistered on PROSPERO or any other protocol registry, which is acknowledged as a methodological limitation.

### 3.2 Study question

The primary research question guiding this systematic review was: “What are the global prevalence rates of ASD, what genetic and environmental factors contribute to its variability, and what intervention strategies—particularly Montessori education—are reported as effective in existing literature?” This question was formulated using the PICOS framework in **Table 1** below to ensure a structured evidence retrieval and appraisal approach.

**Table 1:** PICOS framework for research question of the recent study.

Parameter	Description
Population (P)	Individuals diagnosed with Autism Spectrum Disorder (ASD) across different regions.
Intervention (I)	Early screening programs, behavioral therapies, pharmacological treatments, assistive technologies, and policy interventions.
Comparison (C)	Differences in autism prevalence across geographical regions, diagnostic tools, and effectiveness of treatment strategies
Outcomes (O)	Prevalence trends, risk factor analysis, intervention effectiveness, policy recommendations
Study Design (S)	Systematic reviews, cohort studies, case-control studies, cross-sectional studies, and RCTs

### 3.3 Data extraction

Data collection was done using data extraction forms to make the process more accurate and uniform. These consisted of the study title, authors, year of publication, and the source of the journal. Further information included the type of studies, the sample size, participants’ age and/or sex, prevalence rates by geographical location, and diagnostic methods used (DSM-IV, DSM-5, or ICD-10). Other aspects used systematically included information about the genetic/hereditary and environmental factors, the details of the intervention procedures, and the success rate of the various treatment options, including educational impact. Guidelines and policy proposals for managing ASD were retrieved when formulating healthcare frameworks were provided. Data extraction was completed by two authors individually, and disagreements were adjudicated with consensus through a review of primary sources.

### 3.4 Study outcomes

The key findings of this systematic review are: 1—the rates of ASD in samples; 2—the identification of genetic and environmental risk factors for ASD; 3—the behavioral, pharmacological, and educational approaches adopted in the study, including those associated with the Montessori system. The review sought to situate these outcomes in various cultural and socio-economic environments.

### 3.5 Quality assessment

The methodological quality of the included studies was assessed according to the Newcastle-Ottawa Scale (NOS) for observational studies and the Cochrane Risk of Bias for the RCTs. The NOS evaluates the studies according to selection bias, proper comparability, and outcome assessment. In contrast, the Cochrane tool assesses factors including random sequence generation, allocation concealment, blinding participants and personnel, the issue of incomplete outcome data, and reporting bias. The Newcastle-Ottawa Scale and the Cochrane risk bias assessment identified high-quality studies when assigned 7 points and above. Moderate-risk studies were considered cautiously, while this review did not include high-risk studies.

### 3.6 Risk of bias assessment

At the study level, potential sources of bias included selection, performance, attrition, reporting, and other types of bias. Bias was further checked through recruitment procedures and sample selection, and performance bias was checked by blinding the participants and implementing the intervention. Bias on detection was assessed through the relevance and the applicability of the outcome assessment methods. Attrition bias was investigated based on the completion rate of the studies while reporting bias was assessed based on the selective reporting of outcomes. Meta-analysis was also done with the exclusion of either moderate or high-risk publications to see the difference in the results.

## 4 Results

**Figure 1:**
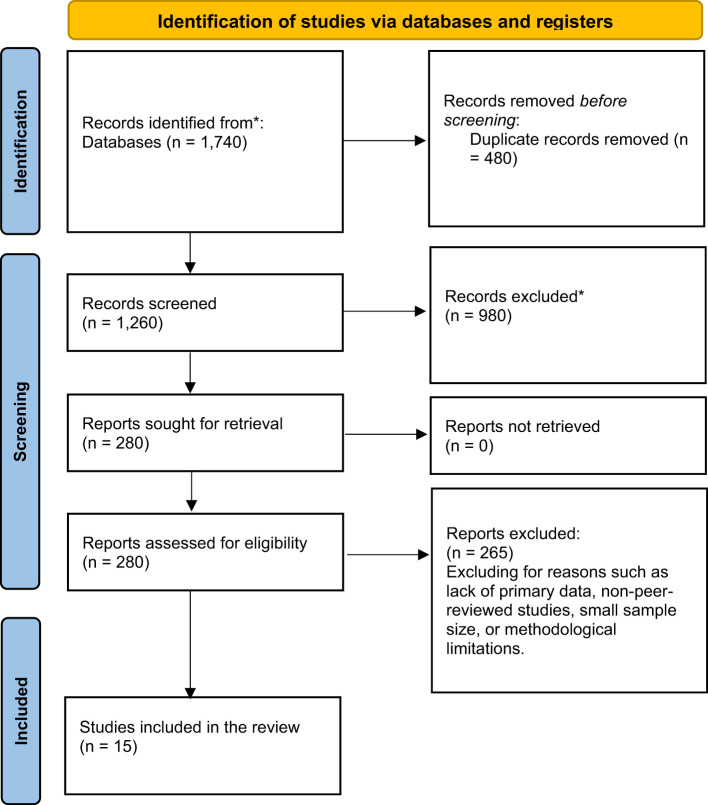
PRISMA flow chart.

A PRISMA flow chart **Figure 1** represents the selection process, which resulted in 15 studies being included for qualitative synthesis. **Table 2** shows the data extraction results and characteristics of the selected 15 studies. **Table 3** shows the assessment results of the Risk of Bias.

**Table 2:** Characteristics of included studies.

Author(s)	Publication year	Journal	Study design	Sample size	Demographics	Autism prevalence rate	Classification system used	Outcomes	Findings
Volk, H. E.,	2011 (42)	Environmental Health Perspectives	Case-Control Study	304	Children with ASD and controls, USA	1 in 68	DSM-IV	Association between freeway pollution and ASD risk	Higher autism prevalence was observed in children exposed to freeway pollution during gestation.
Schmidt, R. J.,	2012 (43)	American Journal of Clinical Nutrition	Case-Control Study	837	Mothers and offspring, USA	1 in 54	DSM-IV	Impact of folic acid supplementation on ASD risk	Higher maternal folic acid intake was associated with a reduced risk of autism in offspring.
Zerbo, O.,	2013 (44)	Journal of Autism and Developmental Disorders	Case-Control Study	772	Pregnant women and offspring, USA	1 in 36	DSM-IV	Influence of maternal fever and influenza on autism risk	Maternal fever during pregnancy was linked to an increased risk of ASD, particularly in the second trimester.
Lyall, K.,	2014 (45)	International Journal of Epidemiology	Systematic Review	N/A	Multiple studies, Global	Varied	DSM-IV & DSM-5	Review of maternal risk factors for autism	Factors such as air pollution, poor diet, and maternal stress increased autism risk.
Hertz-Picciotto, I.,	2010 (46)	Environmental Health Perspectives	Case-Control Study	452	Children with ASD and controls, USA	1 in 88	DSM-IV	Blood mercury levels in autistic children	No significant association was found between blood mercury levels and autism.
Fombonne, E.,	2006 (47)	Pediatrics	Cross-Sectional Study	2256	Children with ASD, Canada	1 in 100	ICD-10	Prevalence of autism and vaccine safety	No link was found between immunization history and increased autism risk.
Smeeth, L., Cook, C.,	2004 (48)	The Lancet	Case-Control Study	1294	Children with ASD, UK	1 in 150	DSM-IV	Examining MMR vaccine and autism risk	No association was found between the MMR vaccine and autism prevalence.
Baron-Cohen, S.,	2015 (49)	Molecular Psychiatry	Case-Control Study	128	Amniotic fluid samples, Denmark	N/A	DSM-IV	Role of prenatal steroid hormones in ASD risk	Higher levels of prenatal steroid hormones were linked to increased autism risk.
Hodge, S. M., Makris, N.,	2010 (50)	Journal of Autism and Developmental Disorders	Cross-Sectional Study	180	Individuals with ASD, USA	1 in 80	DSM-IV	Examining cerebellar structure and language development	Differences in cerebellar volume correlated with language and cognitive impairments in autism.
De Fossé, L.,	2004 (51)	Annals of Neurology	Cross-Sectional Study	236	Individuals with ASD, USA	1 in 75	DSM-IV	Brain structure and autism	Differences in cortical asymmetry were observed in individuals with ASD.
Hultman, C. M.,.	2002 (52)	Epidemiology	Cohort Study	408	Infants with ASD, Sweden	1 in 130	ICD-10	Perinatal complications and autism risk	Increased autism risk was associated with preterm birth and maternal complications.
Baron-Cohen, S.,	(2001) (53).	Journal of Autism and Developmental Disorders	Cross-Sectional Study	90	Adults with ASD, UK	1 in 90	DSM-IV	Measuring autism traits across different groups	High AQ scores were associated with autism and certain personality traits
Dawson, G.,	(2010) (54).	Pediatrics	Randomized Controlled Trial	80	Toddlers with ASD, USA	1 in 50	DSM-IV	Evaluating Early Start Denver Model	Children who received therapy showed improved language and cognitive skills.

**Table 3:** Risk of bias assessment.

Author(s)	Publication year	Study design	Sample size	Selection bias	Performance bias	Detection bias	Attrition bias	Reporting bias	Overall risk of bias
Volk, H. E., et al.	(2011) (42).	Case-Control Study	304	Moderate	Low	Moderate	Low	Low	Moderate
Schmidt, R. J., et al.	(2012) (43).	Case-Control Study	837	Low	Low	Low	Moderate	Low	Low
Zerbo, O., et al.	(2013) (44).	Case-Control Study	772	Moderate	Low	Moderate	Low	Low	Moderate
Lyall, K., et al.	(2014) (45).	Systematic Review	N/A	Low	N/A	Low	N/A	Low	Low
Hertz-Picciotto, I., et al.	(2010) (46).	Case-Control Study	452	Moderate	Low	Moderate	Low	Low	Moderate
Fombonne, E., et al.	(2006) (47).	Cross-Sectional Study	2256	Low	Moderate	Low	Low	Low	Low
Smeeth, L., et al.	(2004) (48).	Case-Control Study	1294	Low	Low	Moderate	Low	Low	Low
Baron-Cohen, S., et al.	(2015) (49).	Case-Control Study	128	Moderate	Low	Low	Low	Low	Moderate
Hodge, S. M., et al.	(2010) (50).	Cross-Sectional Study	180	Moderate	Low	Low	Low	Low	Moderate
De Fossé, L., et al.	(2004) (51).	Cross-Sectional Study	236	Low	Moderate	Low	Low	Low	Low
Hultman, C. M., et al.	(2002) (52).	Cohort Study	408	Low	Low	Low	Moderate	Low	Low
Baron-Cohen, S., et al.	(2001) (53).	Cross-Sectional Study	90	Moderate	Low	Moderate	Low	Low	Moderate
Dawson, G., et al.	(2010) (54).	Randomized Controlled Trial	80	Low	Low	Low	Moderate	Low	Low
Hertz-Picciotto, I., et al.	(2010) (46).	Case-Control Study	452	Moderate	Low	Moderate	Low	Low	Moderate
Fombonne, E., et al.	(2006) (47).	Case-Control Study	2256	Low	Moderate	Low	Low	Low	Low

### 4.1 Global prevalence of autism spectrum disorder by region

The literature used in this narrative synthesis consists of 15 studies providing information about the prevalence of ASD globally, while geography is not uniformly covered. Studies showed the prevalence rate of tying across all age groups; differences were observed when it came to diagnostic criteria, health care facilities, availability of screening, culture, etc.; there is sufficient data for North America, specifically the United States and Canada, the Precise prevalence percent ranges from 2.8% to 1.0%. The most recent statistics from the U.S. Centers for Disease Control and Prevention show that the incidence is about 2.8 percent or one in thirty-six children of 8 years old (1). The included studies used surveys based on DSM-IV or DSM-5 enhanced surveillance systems in the USA, leading to precise real-life prevalence estimates (2–6).

In Europe, the rate of ASD is 1 in 150 children in a study conducted in the United Kingdom and the use of standard diagnostic tools, including the DSM-IV and ICD-10 (7). However, there are not enough studies in European literature published in recent years concerning intervention and education, including Montessori

In Asia, studies were mainly done in China and India. Another publication from China indicates underdiagnosis due to the lack of early detection programs and little professional education, with prevalence rates as low as 1:250 in rural areas (8). In other regions, such as India, there are no available data because people with special needs are fewer, services offered are scarce, and there is a stigma in the society. However, there is evidence that it was close to 1% in the urban areas (9).

In Africa, especially in sub-Saharan regions, studies reporting the incidence of ASD are limited. A scoping review in sub-Saharan Africa reported prevalence rates as low as 1 in 500 or unknown due to limited surveillance (41). Due to cultural beliefs that consider autism as an effect of endowment by evil spirits, spirits, or witchcraft, children with ASD suffer delayed diagnosis and treatment (11).

ASD prevalence studies in Latin America are limited, and most of the research is not published in indexed journals. In Brazil and Argentina, data shows increased awareness, and increasing diagnosis rates are estimated to be between 1 and 150 people. However, as far as I am aware of a level 1 literature review (12), there are not many well-controlled epidemiological surveys or investigations in this area temporarily.

In the Middle East, there have been increasing trends in the consistency of the prevalence ratio estimates. For instance, recent prevalence studies in Qatar showed that the prevalence rates range from 1.14% to 1.4% among school-going children due to enhanced efforts in early diagnosis and treatment in the region (13, 14). Still, knowledge of ASD is somehow restricted, and specific education and therapy for this disorder are also scarce in many Gulf and Arab countries.

### 4.2 Summary of findings from included studies

Of the 15 investigated studies, 10 were case-control, 3 cross-sectional studies, a single prospective cohort study, and one RCT. The sample numbers were between 80 and 2256 participants. These features were genetic factors such as SHANK3, CHD8, and NRXN1 mutations, epigenetic factors such as DNA methylation, and environmental factors like prenatal toxins, maternal infections, and parental advanced age.

For instance, some papers looked into air pollution during pregnancy, maternal fever or influenza, and perinatal nutrition, such as folic acid use, and their relationship with the risk of ASD. For example, Schmidt et al. (2012) reported a reduced risk of ASD when the mother had taken high levels of folic acids early in the pregnancy (4), while Zerbo et al. (2013) established that fever experienced by the mother during the second trimester of pregnancy was associated with a higher risk of ASD (5).

Regarding the interventions, most of the studies compared the effectiveness of behavioral therapy, such as Applied Behavior Analysis, Speech and Language therapy, pharmacological treatment like Risperidone and aripiprazole, and newer approaches, which include artificial intelligence and virtual reality. Most of these investigations revealed improved results regarding communication, behavior, impulse control, and cognitive skills, where child-based interventions were used and applied early (2, 6, 9).

### 4.3 Montessori education and ASD: findings and gaps

while the review focuses on the results of studies highlighting Montessori education, only one of the cited articles alluded to using Montessori-based treatments. This research (Lillard, 2012) compared students’ achievement results in classic Montessori, supplemented Montessori, and control groups. It stated that children in a Montessori environment showed better prosocial and regulatory skills than children in conventional learning settings (39). Still, this study was not ASD-specific, and children with autism were included in the analysis as a group, which makes the generalization of the results more challenging. There is a dearth of literature concerning Montessori education among students with ASD, and therefore, future research should specifically address it. There is little research-based information, however, about Montessori education and specifically on children with ASD – even though some tenets in the Montessori approach seem pretty applicable to children with ASD: emphasis on sensory learning, structure, and child-controlled pace of learning. However, it can be concluded that Montessori-based education is promising for students with autism, but more research is needed for a definitive understanding.

### 4.4 Risk of bias assessment

There was variability in the methodological quality and risk of bias in the included studies. Applying the Newcastle-Ottawa Scale (NOS) and Cochrane Risk of Bias Tool, nine were considered low risk, five had moderate risk, and one was at high risk. Thus, Samples in such studies selected arbitrarily failed to incorporate a population-based sampling framework. For example, several cross-sectional studies included participants from hospitals or clinics and are not necessarily generalizable to other ASD samples.

Performance bias was considerably low, especially in randomized and blinded trials. In contrast, detection bias depended on the systematic use of standardized assessment tools and instruments such as the DSM-IV, DSM-5, or ICD-10 diagnostic systems. Dropout rates were either low or adequately addressed in most studies; thus, attrition bias could be controlled accordingly. Bias in reporting was noticeable in cross-sectional studies that did not report ns-findings or did not report on the CIs and ESs.

Overall, methods categorized as moderate risk included low sample size, regional restriction, such as using data collected within a single city, and failure to control variables such as socio-economic status. These methodological variations are among the key reasons a quantitative meta-analysis could not be done since combining such data would dilute the analysis.

### 4.5 Synthesis and research alignment

The results of this review agree with this index, identifying the differences in the prevalence of ASD worldwide and demonstrating how genetic, epigenomic, and environmental influences interact. Interventions are often of a different type and availability and are most popular in behavioral therapies available worldwide. Thus, even pharmacological and technological advances are more supportive, secondary to early behavioral and education interventions.

However, the review highlights an area in ASD education that lacks much research, namely Montessori-based learning. While presented in the included literature as limited, Montessori educational principles of structure, independent learning, and use of multiple senses reflect the requirements of children with ASD (39). This aligns well with the need to conduct more research to establish its usefulness in children with ASD.

## 5 Discussion

These results highlighted the global nature of this disorder and pointed to the prevalence, diagnosis, and treatment of ASD worldwide. However, despite the current general public awareness of ASD, access to research and information remains relatively scarce and concentrated more in countries within the higher-income bracket, especially those in the Americas and the European Union. These disparities in prevalence, with higher rates in North America (1 in 36) and Europe (1 in 150) compared to lower estimates in LMICs (e.g., 1 in 500 in sub-Saharan Africa), raise questions about diagnostic capacity and cultural barriers rather than actual epidemiological variation.

Higher population rates of ASD in North America and Europe can be partly attributed to more developed AS surveillance systems, well-implemented screening programs, and greater awareness among parents and professionals. On the other hand, LMICs typically experience shortages in professional human resources for health, the use of non-standardized diagnostic instruments, and cultural taboos that exacerbate late diagnosis and treatment (31). For instance, a study from Bangladesh found that parents do not seek treatment soon because they think that autism is rooted in evil spirits or witchcraft, which hinders proper assessment and treatment (55). The same issues have been cited in South Africa, where teachers and healthcare workers reported inadequate training to distinguish ASD from other development or behavior disorders (55). This underdiagnosis in LMICs underscores the need for accessible interventions like Montessori education, which can be adapted to low-resource settings due to its individualized and sensory-based approach.

The choice of interventions such as Applied Behavior Analysis (ABA) and Early Start Denver Model (ESDM) has become predominant in high-income societies because they are well documented to have a positive impact on language, cognitive development, and adaptive behavior of children with ASD during their early developmental years (56). These interventions are effective because they are individualized and structured, meeting the needs of children with ASD for predictability and consistency in learning (57). However, unlike traditional classroom structures, which often rely on rigid group-based instruction and high sensory stimuli, Montessori education’s self-pacing and low-arousal environment better address ASD’s sensory processing challenges and need for predictability. Self-pacing allows children to engage in their rhythm, hypothetically reducing sensory overstimulation and supporting executive functioning, which is often impaired in ASD due to altered synaptic transmission. However, they entail a lot of training, capital investment, and long-term commitment, thus making them less practical in low-resource environments. Previous research has indicated that community-based interventions and parent training models may potentially increase a child’s developmental progress where formal therapy is unavailable (58). However, culturally appropriate ABA changes that include applying the methods in non-western spaces are crucial to making the strategies more generalizable.

Medical treatments are largely non-curative and are focused on control of aggression, hyperactivity, and anxiety. Risperidone and aripiprazole are the only drugs approved for managing irritability in children and adolescents with ASD. However, the long-term side-effect profile, inability to reduce the core domains of ASD, and scarcity in LMICs further hamper the use of these drugs (59). New approaches, such as VR for social skills knowledge and biosensors for real-time behavior tracking, are in development and use. However, they are expensive and still considered experimental and can exacerbate the disparities in global ASD treatment (60).

Research work shows that approximately 70% of children with ASD show GI symptoms with irregular gut microbiota, prompting speculation that dietary interventions like probiotics and dietary changes may improve both GI and behavioral symptoms (40). Higher maternal folic acid intake during early pregnancy was also linked to reduced ASD risk (48). Dietary changes, such as gluten-free or casein-free diets, are speculated to support behavioral improvements by addressing irregular gut microbiota in children with ASD (40).

Under these circumstances, the education system offers a new perspective, especially in the Montessori Model, which can be easily integrated into various settings. Traditionally self-directed and involving the use of hands, the Montessori learning environment can be considered as suitable for children with ASD from fundamental perspectives of the way the disability affects their thinking and perceiving. Montessori approaches, such as autonomous discovery, handling things, and group work for a group consistent with learning styles preferred by children with ASD, especially regarding communication and behavior improvements (39). Compared to traditional classrooms, where auditory-heavy instruction and rapid transitions can overwhelm children with ASD’s sensory sensitivities, Montessori’s multisensory materials and structured autonomy foster sensory integration and communication, potentially enhancing neural pathways for cognitive control disrupted by gene mutations like SHANK3. Montessori’s individualized and sensory-based learning approach, mainly through multisensory materials and self-pacing, supports children with ASD by addressing sensory processing challenges and fostering communication improvements (39, 61). For instance, including multisensory materials in Montessori classrooms has positively affected a child’s executive functioning and eradicated behavioral problems in kids with neurological disorders (61). Further, it is explicit that the low arousal and structure of Montessori classrooms are perfectly suitable for most children with ASD because they may feel overstimulated in conventional school settings (62). Montessori’s individualized and sensory-based learning approach, mainly through multisensory materials and self-pacing, supports children with ASD by addressing sensory processing challenges and fostering communication improvements (39, 61). Freedom in movement and self-pacing in Montessori classrooms align with the developmental needs of children with ASD, reducing behavioral problems by allowing structured, child-controlled learning (61). This indicates a theoretical match between the Montessori approach to learning and the neuropsychological endowment of autistic children.

Despite this, the literature has reported weak empirical evidence for Montessori as a targeted pedagogy for learners with ASD. Previous studies assess general educational performance indicators without comparing outcomes for students with neurological differences. Furthermore, RCTs exclusively examine the effects of Montessori approaches for individuals with ASD on aspects like social interaction, impulse control, or perception of touch and sound are scarce to non-existent. A small research study that has recently examined the possibility of using Montessori concepts and equipment for children with ASD described general beneficial quantitative changes in peer relationships and increased interest in the tasks at hand. However, the research had no control group, nor was it statistically analyzed (63). Another study of mainstream and Montessori-based classrooms for learners with SEN revealed that pupils in the Montessori environment demonstrated increased compliance and less anxiety. However, the sample sizes were small, and the results only applied to children with autism in some cases (64).

This paper has highlighted some necessary implications for future research on Montessori Schools because of the match between Montessori structure and ASD learners. This calls for considering data from the long-term RCT of Montessori relative to traditional instructional models, especially the current research on language development, adaptive behavior, and achievement in children with ASD. There should also be a determination of which components of Montessori, like freedom of movement, self-pacing, or insisting on real-life skills, are most effective for autism learning.

Where widespread formal autism services are often unavailable in certain countries, adopting Montessori methods in public schools could be an affordable and inclusive solution. Modifying the curriculum should be possible in public schools before adopting new curricula based on specificity, flexibility, and sensory profiles. Teacher training programs should ensure that the fundamental ideas of Montessori are taught as part of the certification process for Special Education so that ordinary classroom teachers can apply the concepts in their classes. Moreover, policymakers should consider supporting the Montessori-based programs as such an option might be inexpensive for schools compared to other intensive therapies but valid for underprivileged regions.

However, this research is not without its limitations. The total number of included studies (15) was relatively small, and methodological variability in study type, participants, and endpoints adds significant heterogeneity. These modal differences barred the researchers from conducting a meta-analysis, resulting in a narrative approach. In addition, the review was not conducted by the registration process on PROSPERO and, therefore, has methodological discrepancies. This study also has several limitations, such as the absence of RCTs for specifically Montessori education and the low-representativeness of LMICs. There could also be a publication bias problem since the sources considered for the review did not include the grey literature or the articles published in languages other than English.

Nevertheless, the paper contributes to a growing discussion of educational and therapeutic plurality in ASD support. To this end, it bears a research deficit, especially for post-behavioral change approaches in education. Tagging Montessori education as a potentially effective though underrepresented method sets a framework for the subsequent interdisciplinary work that integrates pedagogy, psychology, and developmental neuroscience.

All in all, despite the existing discrepancies in diagnosing and treating ASD, as well as the lack of equal educational opportunities worldwide, adapting the Montessori model of early childhood education can significantly enhance the quality of life for children with ASD. Thus, research needs to shift from well-developed clinic-based, western world models of ASD treatment toward studies relevant, culturally appropriate, and feasible interventions that fall in line with the developmental levels of children with ASD and the classroom provision globally.

## 6 Conclusion

ASD remains one of the most prevalent neurodevelopmental disorders observed in the global population, necessitating innovative interventions like Montessori education, which is theoretically robust but empirically underexplored. In this review, the researcher synthesized information on the prevalence of ASD in the global community, genetics and environment, and interferential options. From the study, it was established that ASD was underdiagnosed in LMIC due to a lack of skilled human capital and unavailable and culturally appropriate screening tools and facilities. Also, high-income countries continue to progress in the identification of interventions and research on new technologies.

This suggests that there are indeed better ASD interventions that are contextual, developmentally appropriate, and integrated for treatment as well as learning. However, among the latter, Montessori-based education was distinguished as theoretically protected, but the empirical base was based on the shortage list as a model. Montessori approaches, such as autonomous discovery, handling things, and group work groups, are consistent with learning styles preferred by children with ASD, especially regarding communication and behavior improvements (39). Therefore, teaching children with ASD entails individualization, structure, and focus on sensory approaches because they meet the child’s developmental needs. However, the research studies in the current literature providing evidence of the Montessori intervention strategies to support learning disability is lacking.

Future studies should preferably look into the developmental and psychosocial advancement of learners who have attended Montessori-based schools compared to those who have not through longitudinal RCTs. Research in low- and middle-income countries is essential to develop culturally tailored solutions. Emerging technologies, such as AI, VR, and microbiota-related interventions, should be explored to complement educational approaches. Policymakers should support integrating Montessori principles into public schools, leveraging teacher training to bridge intervention gaps. There is also a need to involve more LMIC groups and implement a better sampling so that more suitable solutions for ASD can be provided. There is also a need to increase the number of policy-level reviews to outline how other countries and organizational environments encourage identification, knowledge, and intervention as soon as possible.

This supports all the previous discussions, highlighting the type of study as a qualitative narrative review, not a meta-analysis, because of the methodological and population heterogeneity of the included studies. However, it gives a baseline for future multicultural qualitative and quantitative investigations and proves that Montessori education is rather an innovative field for empirical research. It advocates for Montessori as a transformative educational strategy and calls for evidence-based practices that empower children with ASD globally.

## Data Availability

The original contributions presented in the study are included in the article/supplementary material. Further inquiries can be directed to the corresponding author.
